# Shengjiang Powder alleviates oxidative stress damage and fibrosis in mice with atherosclerosis concurrent with non-alcoholic fatty liver disease

**DOI:** 10.1186/s41065-025-00598-y

**Published:** 2025-11-12

**Authors:** Jia He, Bingjiu Lu, Yan Zhang, Jingran Sun, Bo Fu, Tianqing Wang

**Affiliations:** 1 Liaoning University of Traditional Chinese Medicine, Shenyang, China; 2 Shenyang Hospital of Traditional Chinese Medicine, Shenyang, China; 3 Affiliated Hospital of Liaoning University of Traditional Chinese Medicine, Shenyang, China; 4 The Second Hospital of Liaoning University of Traditional Chinese Medicine, Shenyang, China

**Keywords:** Non-alcoholic fatty liver, Shengjiang powder, Atherosclerosis, Inflammation fibrosis

## Abstract

**Objective:**

To investigate the effects of “Shengjiang Powder”, a representative formula for “simultaneous treatment of liver and heart,” on liver tissue inflammation and fibrosis in mice with atherosclerosis(AS) concurrent with non-alcoholic fatty liver disease (NAFLD).

**Methods:**

Ten wild-type male C57/B6J mice were assigned to the control group, and 40 ApoE -/- mouse were randomly divided into the model group, atorvastatin group, and traditional Chinese medicine (TCM) treatment groups. The model group, atorvastatin group, and TCM treatment groups were fed a high-fat Western diet for 12 weeks. Atorvastatin and TCM groups were administered via gavage, while the control group and model group received sterile purified water via gavage for 12 weeks. Serum levels of ALT, AST, TRIG, TC, LDL, as well as liver tissue levels of SOD, MDA, and GSH were measured. HE staining was used to evaluate liver tissue morphology and inflammatory infiltration. Western blot was used to detect the effect of Shengjiang Powder on the activation of AMPK/mTOR signaling pathway. Network pharmacology analysis was performed beforehand to identify potential targets of Shengjiang Powder in regulating fatty liver and atherosclerosis, with AMPK identified as a key target.

**Results:**

Compared with the model group, the Shengjiang Powder treatment reduced serum levels of TRIG, TC, and LDL (*P* < 0.05), increased liver SOD and GSH activity (*P* < 0.01), decreased MDA (*P* < 0.01), alleviated liver steatosis, reduces the area of aortic sinus plaques, improved hepatic steatosis and inflammation, inhibited the expression of inflammatory factors and activated the AMPK/mTOR signaling pathway, consistent with the network pharmacology prediction that AMPK is a critical regulatory target.

**Conclusion:**

Treatment with “Shengjiang Powder,” a representative formula for “simultaneous treatment of liver and heart,” can slow the progression of atherosclerosis and concurrent NAFLD. The dosage shows a positive correlation with efficacy, and this effect is related to the regulation of liver oxidative stress and inflammation-induced fibrosis pathways.

**Supplementary Information:**

The online version contains supplementary material available at 10.1186/s41065-025-00598-y.

## Introduction

“Non-alcoholic fatty liver disease (NAFLD) and atherosclerosis (AS) are both chronic metabolic diseases characterized by high clinical incidence, difficult prevention and treatment, and long-term significant harm. NAFLD is the most prevalent chronic liver disease [1], while AS represents the leading cause of death from cardiovascular diseases [2]. Both conditions share similar risk factors, pathological bases, and developmental inducements, often occurring concurrently. Numerous meta-analyses and epidemiological evidence have demonstrated a significant association between their pathogenesis [3–7]. Given the notably increasing comorbidity ratio, the clinical field has consistently sought a safe, reliable, effective, and affordable treatment regimen that can simultaneously benefit the prevention and treatment of both diseases. Although the primary drugs currently used to treat AS, such as statins, have shown some efficacy in managing NAFLD [8], they are not without issues, including a single target mechanism and potential side effects, particularly liver injury. In our clinical practice, we have observed that the “simultaneous treatment of liver and heart” formula can effectively lower blood lipids and ameliorate the progression of fatty liver in patients with AS.

“Co-morbidity of liver and heart diseases” is an extension and expansion of the traditional Chinese medicine (TCM) principle of “treating different diseases with the same method,” embodying the holistic dialectical perspective and treatment strategies of TCM [9, 10]. Liver diseases can affect the heart vessels, leading to vascular endothelial damage. Although they involve different organs, they are physiologically closely connected through meridians and blood vessels, and their pathologies mutually influence each other. The theory of “simultaneous treatment of liver and heart” is proposed under the guidance of the TCM principle of treating different diseases with the same method, based on the close physiological relationship between the heart and liver and the shared pathological foundation of their diseases [11, 12]. The theory of combined liver and heart diseases represents an innovation and development of the holistic thinking in TCM. Ancient TCM texts do not specifically record the disease names of metabolic dysfunction-associated fatty liver disease (NAFLD) combined with atherosclerosis (AS). Our research has found that it can be categorized under the TCM concepts of “liver stagnation” and “chest obstruction”. Based on the principle from “Xue’s Medical Cases” that “when liver qi is smooth, heart qi is harmonious; when liver qi is stagnant, heart qi is deficient,” and combined with years of clinical experience, our team proposes that according to the physiological characteristics of “liver-blood-heart-vessel” and “harmony between liver, heart, and qi” in TCM, it falls within the category of “co-morbidity of liver and heart diseases,” and the treatment principle is “simultaneous treatment of liver and heart”. We believe that “qi mechanism dysfunction” is the key to the development of NAFLD combined with AS. Treatment should focus on soothing the liver and strengthening the spleen in the early stage, protecting the liver and kidney in the middle stage, and addressing phlegm-dispelling and blood-stasis removal throughout the process, providing ideas and methods for the clinical prevention and treatment of NAFLD combined with AS. Therefore, this study intends to construct a mouse model of AS combined with NAFLD, administer different doses of Shengjiang Powder through gavage to obtain in vivo evidence and possible mechanisms of the effective intervention of such drugs on the progression of NAFLD-related inflammation and fibrosis associated with AS, and compare its efficacy with the currently mainstream drug for treating AS, atorvastatin, to explore the advantages of TCM treatment.

## Materials and methods

### Network pharmacology analysis

Chemical compound data was sourced from the HERB database, a high-throughput platform rooted in experiments and references for traditional Chinese medicine (TCM), which includes all four medicinal herbs examined in this research (http://herb.ac.cn/Contact/). Target genes related to TCM components, herbs, and formulations were pinpointed by comparing the chemical fingerprint similarity between TCM ingredients and known drugs. Recognized fatty liver-associated targets were collected through a search of the GeneCards database. Cytoscape (version 3.10.0) was used to build a network that depicts the interaction between herbs and the fatty liver-related targets of Shengjiang Powder. To clarify the biological processes (BP) through which potential Shengjiang Powder targets participate in fatty liver, gene ontology (GO) enrichment analysis was performed. Moreover, Kyoto Encyclopedia of Genes and Genomes (KEGG) pathway enrichment analysis was conducted to gain a thorough understanding of the mechanisms behind Shengjiang Powder’s impact on fatty liver. AutodockVina 1.2.2, an in silico protein-ligand docking tool, was utilized to assess the binding affinities and interaction patterns between candidate compounds and their targets. The molecular structure of emodin, danthron, curcumin was retrieved from PubChem Compound (https://pubchem.ncbi.nlm.nih.gov/), and the 3D coordinates of AMPK (PDB ID: 3AQV) were obtained from the Protein Data Bank (PDB) (https://www.rcsb.org/). In preparation for docking simulations, all protein and ligand files were converted to PDBQT format; this involved removing water molecules and adding polar hydrogen atoms. The grid box was positioned to cover each protein’s domain, enabling unrestricted molecular movement during docking. Autodock Vina 1.2.2 (http://autodock.scripps.edu/) was employed to carry out the molecular docking studies.

### Reagents

Hematoxylin-Eosin Dye (Servicebio, Cat#: G1004-500ML); Oil Red O Dye (Servicebio, Cat#: G1015-100ML); Masson’s Trichrome Dye (Servicebio, Cat#:G1006-100ML); SOD Assay Kit (Solarbio, Cat#:BC0170); MDA Assay Kit (Solarbio, Cat#:BC0025); GSH Assay Kit (Solarbio, Cat#:BC1175); Primary Antibody Rb pAb to LYVE1 (Abcam, Cat#:ab10278); Goat Anti-Rabbit IgG H&L (Alexa Fluor 488) (Bioswamp, Cat#:SAB51361); DAPI (4’, 6-diaminyl-2-phenylindoline dihydrochloride)(Bioswamp, Cat#:DW019); O.C.T. Embedding Medium (Sakura Finetek Japan Co., Ltd., Cat#: 4583); 4% Paraformaldehyde (Servicebio, Cat#: G1101-3 M).

### Animal and disease model preparation

Seven-week-old ApoE^−/−^/C57/B6J mice (specific pathogen-free, SPF grade) were purchased from Beijing SPF Biotechnology Co., Ltd. (Animal Qualification Certificate No.: 202413455). The mice, with a body weight range of 20–30 g, were given one week of adaptive feeding (free access to regular feed and water) before the commencement of modeling. To establish a model of atherosclerosis (AS) combined with non-alcoholic fatty liver disease (NAFLD), the mice were fed a Western high-fat diet purchased from Ruidi Biotechnology (Shenzhen) Co., Ltd. (78.85% regular feed + 21% lard + 0.15% cholesterol). The animal experiments were conducted in accordance with the Guidelines for the Care and Use of Laboratory Animals and approved by the Institutional Animal Care and Use Committee of China Medical University (Shenyang, China; Approval No. CMUXN2023078).

Ten wild-type male C57/B6J mice were assigned to the control group, and 40 ApoE^−/−^ mouse were randomly divided into the model group, atorvastatin group, and traditional Chinese medicine (TCM) treatment groups (low-dose and high-dose Shengjiang Powder groups).

Subsequently, the control group continued to receive a normal diet, while the remaining groups were given a Western high-fat diet for 16 weeks. Starting from the 8th week of the Western high-fat diet administration, each mouse was given a daily intragastric gavage of 0.2 mL per 20 g of body weight. The gavage volume was adjusted weekly based on the average body weight changes of each group. After 8 weeks of continuous gavage, the samples were collected.

### Drug treatment

Composition of Shengjiang Powder: Bombyx Batryticatus (Jiangcan) 10 g, Periostracum Cicadae (Chantui) 5 g, Rhei Radix et Rhizoma (Dahuang) 10 g, Curcumae Rhizoma (Jianghuang) 10 g. All Chinese herbal medicines were purchased from the Pharmacy Department of the Affiliated Hospital of Liaoning University of Traditional Chinese Medicine. Each herbal component was weighed according to the ratio, soaked for 1 h, boiled vigorously, simmered for 20 min, filtered, and decocted three times. The filtrates were combined to make a decoction, which was stored at 4℃ for later use. The daily dosage of raw herbs contained in the Shengjiang Powder decoction for adults was 35 g. According to the “Pharmacological Experiment Methods,” the dosage for mice was calculated based on the equivalent dose ratio converted from the body surface area between humans and animals: the raw herb dosage for mice = 35 g ÷ 70 kg × 70 kg × 0.018 ÷ 0.2 kg = 3.15 g/kg of body weight·d. The calculated daily dosage of raw herbs per kilogram of body weight was 3.15 g/kg of body weight·d, which was used as the medium dose for intragastric gavage of Shengjiang Powder in mice. The dosages for the high-dose and low-dose groups were respectively double and half of this medium dose, with the high-dose group receiving 6.3 g/kg of body weight·d and the low-dose group receiving 1.58 g/kg of body weight·d.

### Body weight and serum biochemical analysis

Starting from the initiation of modeling, the body weight of mice in each group was recorded weekly until the end of drug intervention, which was at the 24th week. At this point, blood was collected from the eyeballs of mice under isoflurane inhalation anesthesia. The whole blood was centrifuged at 4 °C and 3000 rpm for 20 min, and the serum was carefully collected. After diluting with phosphate-buffered saline (PBS) in a 1:2 ratio, the levels of low-density lipoprotein (LDL), triglycerides (TRIG), total cholesterol (TC), glutamic pyruvic transaminase (ALT), millet straw transaminase (AST), and high-density lipoprotein (HDL) were measured. All these measurements were performed using the Beckman Coulter automated biochemical analysis system in the Laboratory Department of Longhua Hospital affiliated to Shanghai University of Traditional Chinese Medicine.

### Histomorphological and pathological examinations

Two liver samples (approximately 0.5 cm × 0.5 cm × 0.3 cm in size, taken from the central part of the largest liver lobe) were immediately placed in 4% paraformaldehyde solution upon extraction. After fixation at 4 °C for 48 h, one liver sample was sequentially dehydrated in 10%, 20%, and 30% sucrose-PBS solutions for 24 h each. The sample was then slightly air-dried and embedded in OCT frozen embedding medium. The maximum cross-section of the aortic sinus was selected, and 8 μm frozen sections of the liver were prepared, taking the largest intact cross-section. The other liver sample was dehydrated and embedded using an automated paraffin dehydration machine, followed by preparation of 5 μm paraffin sections, taking the largest intact cross-section.

### Hematoxylin-eosin staining for detection of liver fatty degeneration

Liver paraffin sections were baked at 60℃ overnight, dewaxed with xylene and graded ethanol to water, stained with HE, washed thoroughly, differentiated with 4% hydrochloric acid ethanol, dehydrated with graded ethanol, cleared with xylene, mounted with neutral balsam, and observed under a microscope.

### Oil red O staining for detection of atherosclerotic plaque size in the aorta and hepatic lipid accumulation

Frozen sections of the aorta and liver were dried at room temperature and fixed with 4% paraformaldehyde. After washing, the sections were stained with Oil Red O working solution, incubated at 37 °C for 2 h, washed, differentiated with 75% ethanol, stained with hematoxylin solution, differentiated with 4% hydrochloric acid ethanol, washed, mounted with gelatin glycerol, and observed under a microscope.

### Detection of oxidative stress damage factors in the liver

Mouse liver samples were frozen in liquid nitrogen. After accurate weighing and thorough homogenization, a 10% liver tissue homogenate was prepared. The supernatant was collected, and the levels of oxidative stress markers (SOD, MDA, GSH) in the liver tissue were measured step by step according to the kit instructions. The OD values were determined using a microplate reader, and the results were calculated based on the formulas provided in the instructions.

### Western blot analysis

Total protein was isolated from liver tissue using radioimmunoprecipitation assay (RIPA) and quantified using the bicinchoninic acid assay (BCA) protein detection kit. The isolated protein was diluted in loading buffer, boiled for 5 min, and then separated by 12.5% SDS-PAGE using 20 µg of protein sample. The separated proteins were transferred onto a polyvinylidene fluoride (PVDF) membrane. The membrane was probed with primary antibodies against TNF-ɑ (dilution 1:1000), IL-6 (dilution 1:1000), AMPK (dilution 1:1000), mTOR (dilution 1:200), p-AMPK (dilution 1:1000), p-mTOR (dilution 1:200) at 4 °C overnight, followed by incubation with horseradish peroxidase-conjugated secondary antibodies at room temperature for 2 h. The protein bands were detected using an ECL advanced system (Millipore) and quantified using Photoshop software.

### Statistical methods

Statistical analysis was performed using SPSS 26.0. Data are presented as mean ± standard deviation. For multiple groups with normally distributed data, one-way ANOVA was used for intergroup comparisons; otherwise, nonparametric tests were employed. A *P*-value < 0.05 was considered statistically significant.

## Results

### Network pharmacology predicts potential pharmacological mechanisms for Shengjiang Powder treatment of fatty liver

**Fig. 1:**
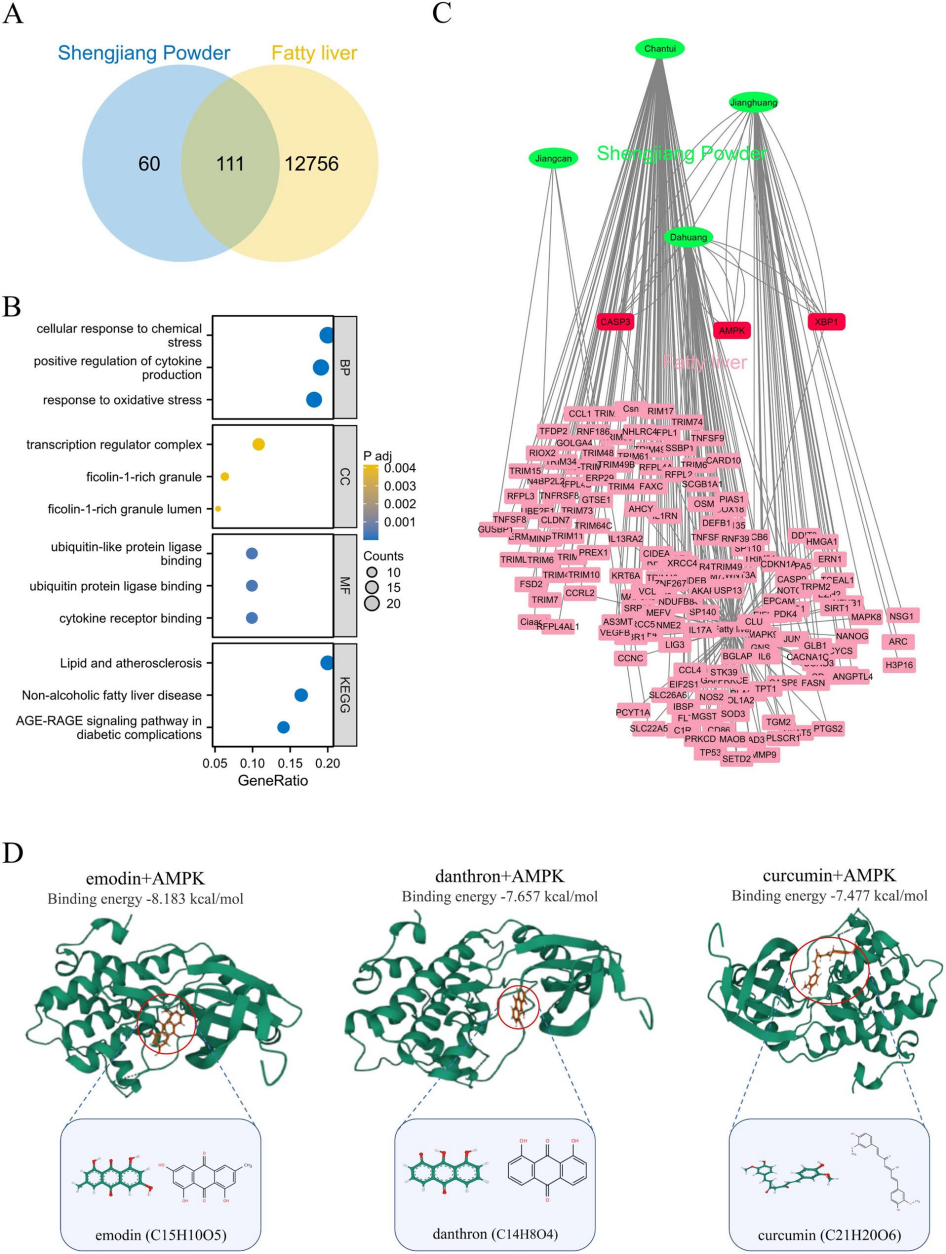
Network pharmacological prediction for Shengjiang Powder treatment of fatty liver. **A**: Venn diagram of the corresponding targets of the effective components of Shengjiang Powder and fatty liver. **B**: GO and KEGG pathway enrichment analysis. **C**: herbs-disease-target networks of Shengjiang Powder against fatty liver. The Green oval nodes represent herbs of Shengjiang Powder, the Red rectangle nodes represent fatty liver genes (≥ 3 effective components targeting the same gene together), and the pink rectangle nodes represent fatty liver genes (≤ 2 effective components targeting the same gene together). **D**: Molecular docking reveals that the three compounds of Shengjiang Powder target AMPK

We retrieved 171 target genes of Shengjiang Powder through predictions using the HERB database. To find target genes shared between Shengjiang Powder and fatty liver, we used a Venn diagram for screening, which resulted in 111 common targets (Fig. 1A). Next, these 111 shared target genes underwent GO and KEGG enrichment analyses. In the biological process (BP) category, the most prominently enriched items were “cellular response to chemical stress” and “positive regulation of cytokine production”. For the cellular component (CC) category, the top enriched terms were “transcription regulator complex” and “ficolin-1-rich granule”. When it comes to the molecular function (MF) category, the most significantly enriched items were “ubiquitin-like protein ligase binding” and “ubiquitin protein ligase binding”. In the KEGG pathway analysis, the top enriched terms were “Lipid and atherosclerosis” and “Non-alcoholic fatty liver disease” (Fig. 1B).

To make the target prediction results easier to visualize and interpret further, we built a compound-target (C-T) network. AMPK, a key molecule that plays a role in both regulating lipid metabolism and adjusting inflammatory responses related to fatty liver, stood out in this network. The C-T network showed that three compounds from Shengjiang Powder target AMPK: emodin, danthron, and curcumin (Fig. 1C). Emodin and danthron come from emodin, while curcumin is obtained from curcumin.

We carried out molecular docking analysis to test the binding affinity between these three Shengjiang Powder compounds and AMPK. Using AutoDock Vina v.1.2.2, we evaluated the binding poses and interactions of these compounds with AMPK, and also calculated the binding energy for each interaction (Fig. 1D). The results showed that all three compounds from Shengjiang Powder formed bonds with AMPK via visible hydrogen bonds and strong electrostatic interactions. What’s more, these compounds successfully filled the hydrophobic pockets of AMPK. In terms of KDR, the binding energies of emodin, danthron, and curcumin were − 8.183, −7.657, and − 7.477 kcal/mol respectively, which points to highly stable binding interactions.

### Effect of Shengjiang Powder on reducing liver weight, liver index, and body weight gain in mice

**Fig. 2:**
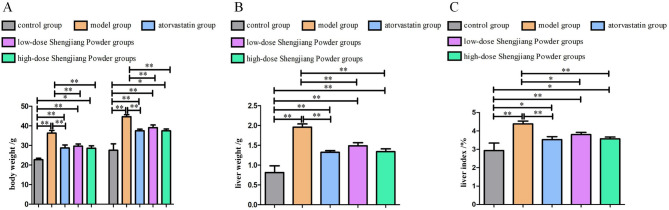
Changes in body weight, liver weight, and liver index in mice. **A**: Changes in body weight before and after drug treatment (16 weeks vs. 24 weeks). Compared to body weight at 16 weeks. **B**-C: Comparison of liver weight and liver index (liver weight/body weight × 100%) after drug treatment

Mice were fed a high-fat western diet starting at 8 weeks of age. Drug treatment was initiated at 16 weeks of age and continued for 8 weeks until 24 weeks of age. After the 8-week drug treatment, the body weight of mice in the model group increased significantly from 12 weeks to 24 weeks (*P* < 0.01, *P* < 0.05) (Fig. 2A). The liver weight in the model group increased significantly, while the high-dose traditional Chinese medicine group showed a significant reduction in liver weight (*P* < 0.01, *P* < 0.05) (Fig. 2B). The liver index (liver weight/body weight × 100%) was higher in the model group compared to the control group, and the high-dose traditional Chinese medicine group showed significant improvement compared to the model group. However, there was no significant difference in liver weight or liver index between the low-dose traditional Chinese medicine group and the model group (Fig. 2C).

### Effect of Shengjiang Powder on improving serum lipid parameters in mice

**Fig. 3:**
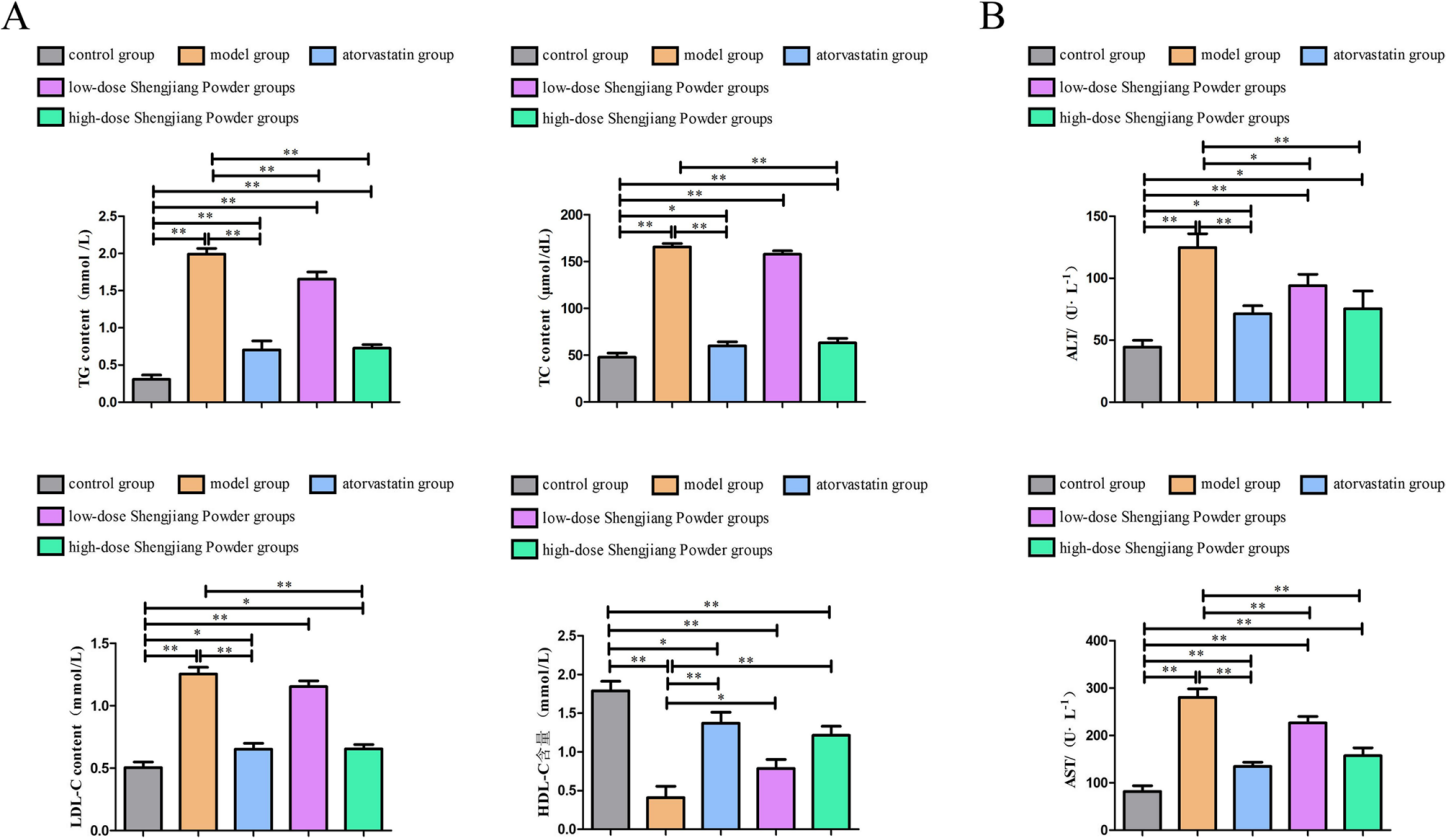
Serum lipids, liver, and kidney function in mice. **A**: Comparison of serum lipid parameters among groups. Compared to the model group. **B**: Comparison of serum liver and kidney function among groups

All four serum lipid parameters were significantly higher in the model group compared to the control group (*P* < 0.01). After intervention with high-dose traditional Chinese medicine and atorvastatin, there was a significant reduction in TRIG levels (*P* < 0.01, *P* < 0.05). Atorvastatin showed a better effect in reducing TC and LDL levels (*P* < 0.01), while the high-dose traditional Chinese medicine group also demonstrated a significant effect (*P* < 0.05). However, the low-dose traditional Chinese medicine did not show any lipid-lowering effects. There were no statistically significant differences in HDL levels among the various interventions. Additionally, high-dose traditional Chinese medicine showed AST-lowering and ALT-lowering effects (*P* < 0.05), while the atorvastatin group showed AST-elevating and ALT-elevating effects (*P* < 0.05). The remaining comparisons between groups were not statistically significant (Fig. 3).

### Effect of Shengjiang Powder on MDA, SOD, and GSH levels in the liver of mice

**Fig. 4:**
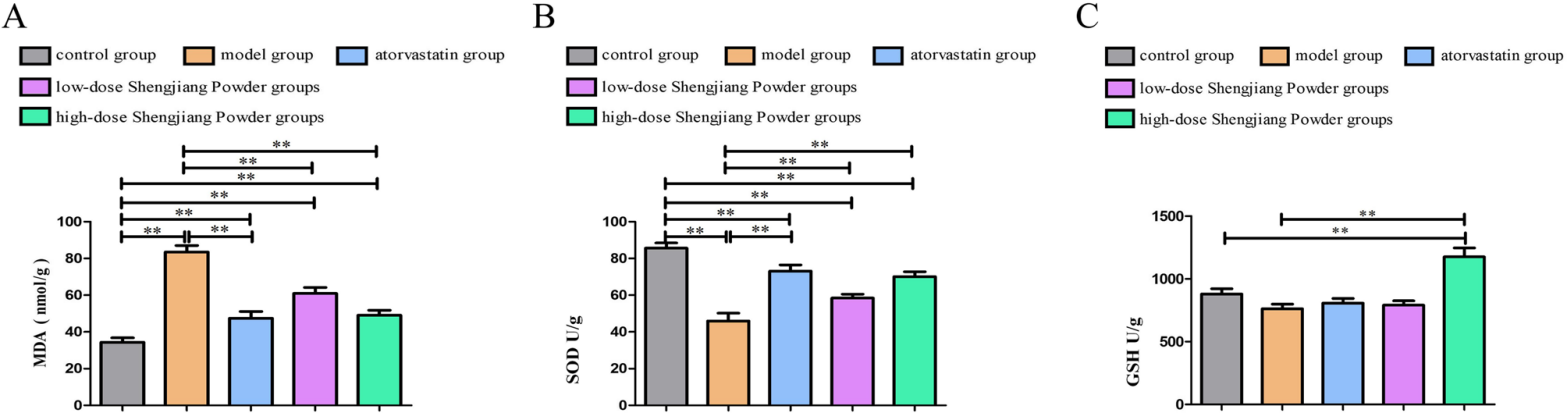
MDA, SOD, and GSH levels in the liver of mice. **A**: MDA levels in liver tissue. **B**: SOD levels in liver tissue. **C**: GSH levels in liver tissue.”

As shown in Fig. 4A, all drug-treated groups significantly decreased MDA levels (*P* < 0.01). Figure 3B demonstrates that the high-dose Shengjiang Powder group significantly increased SOD levels (*P* < 0.05). Although the reduction in GSH activity in the model group compared to the control group was not statistically significant, the Shengjiang Powder intervention treatment group still showed a significant increase in GSH levels in the liver tissue of the model group (*P* < 0.05, *P* < 0.01), as illustrated in Fig. 4C.

### Shengjiang Powder reduces the area of aortic sinus plaques in mice

**Fig. 5:**
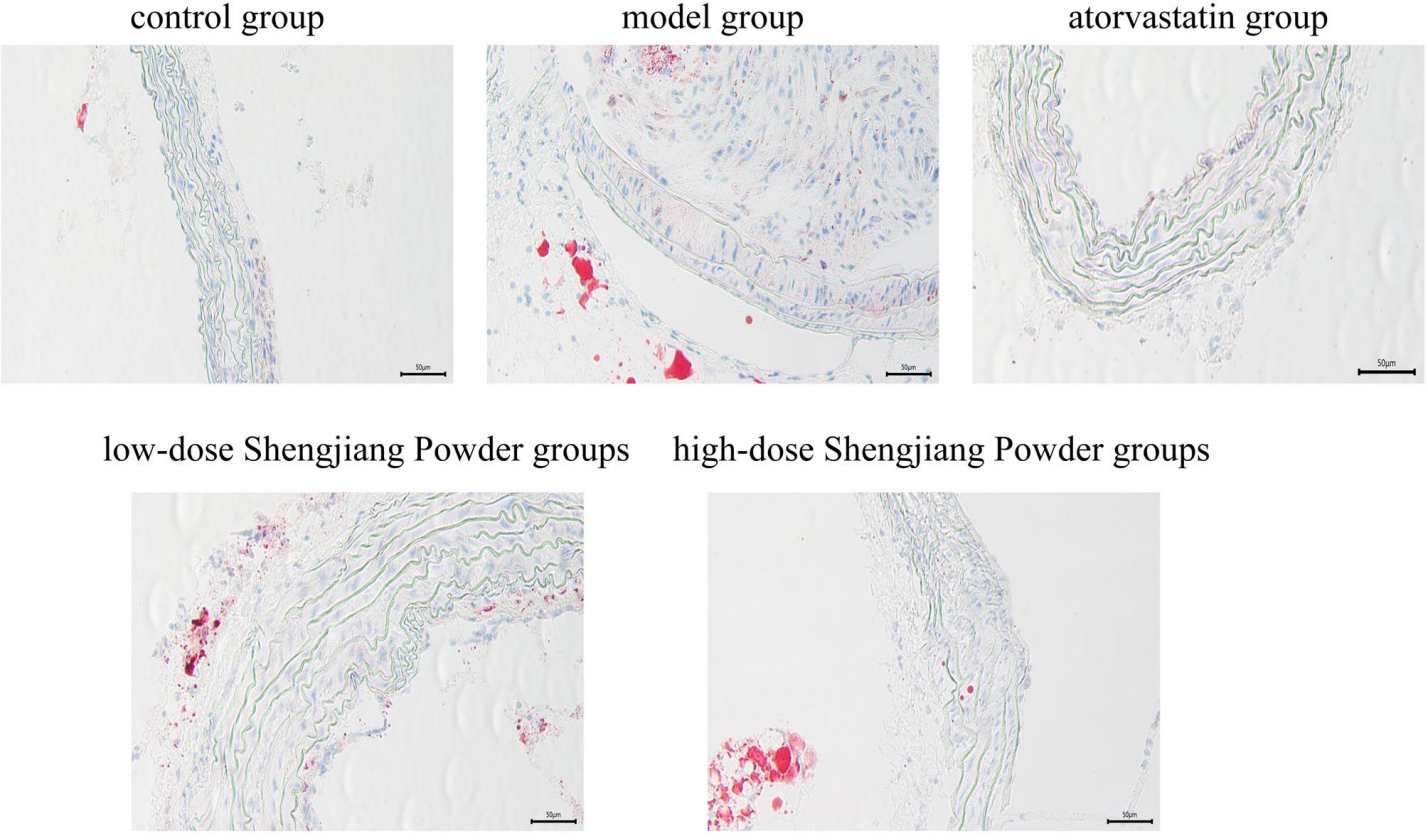
Microscopic changes in aortic sinus of mice with oil red O staining (40× magnification)

AS lipid areas were stained red, nuclei were stained blue-purple, and the interstitium was colorless. The control group showed no significant lipid deposition. The model group exhibited extensive and typical lipid plaques with significant luminal stenosis. The high and low-dose Shengjiang Powder groups improved the plaque area, and the efficacy was positively correlated with the dose. Atorvastatin significantly reduced the lipid extent of the plaques (Fig. 5).

### Shengjiang Powder improves hepatic steatosis and inflammation in mice

**Fig. 6:**
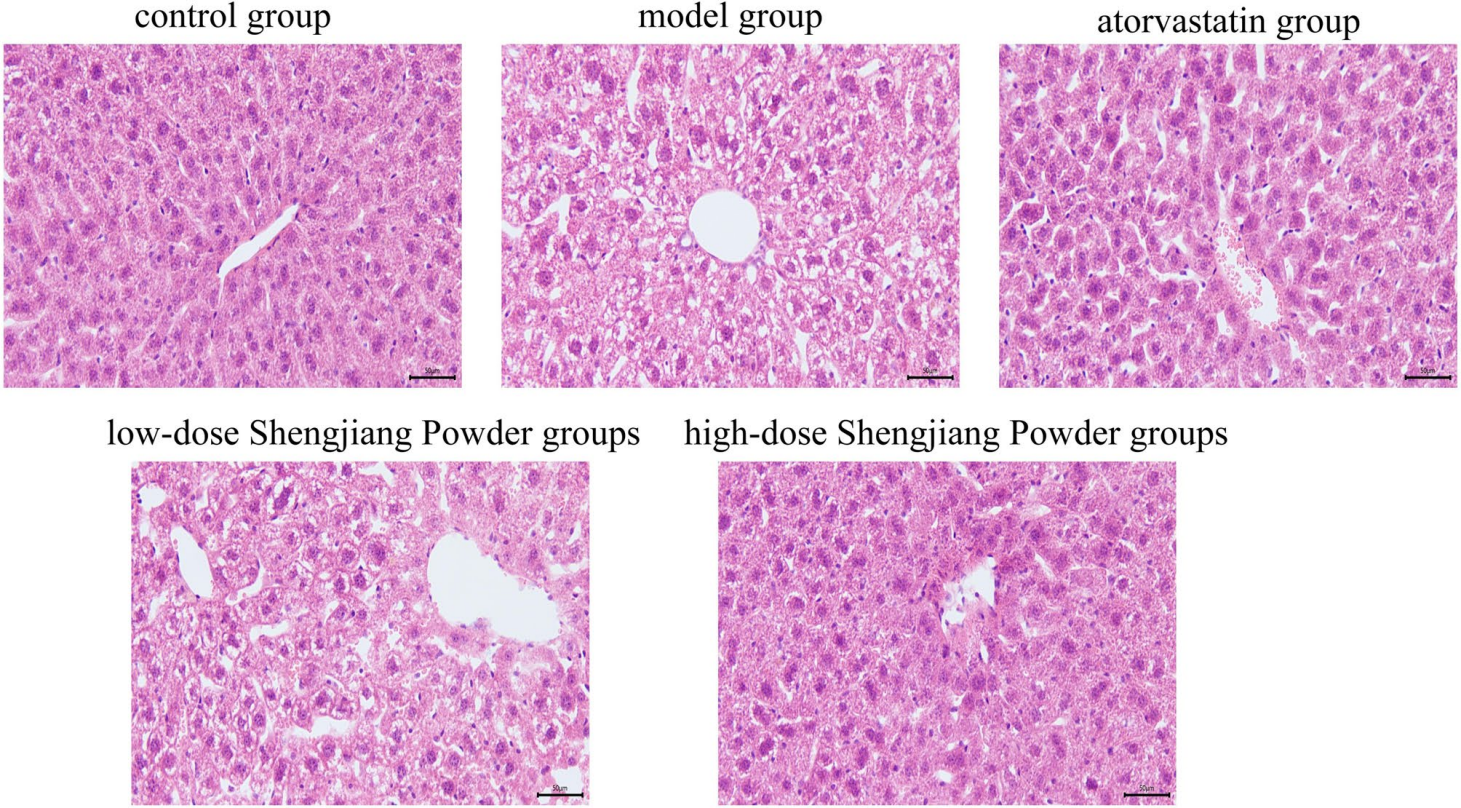
Microscopic changes in mouse liver with HE staining (200× magnification)

Microscopic images of liver HE staining in mice from various groups are presented. The model group exhibited extensive and severe hepatic steatosis (ballooning degeneration) accompanied by increased inflammatory cell infiltration. In the Chinese herbal treatment groups, the extent and severity of hepatic steatosis were reduced in a dose-dependent manner. The atorvastatin group showed a reduction in steatosis, but more inflammatory cell infiltration was observed in the portal area (Fig. 6).

### Shengjiang Powder ameliorates lipid deposition in mouse liver

**Fig. 7:**
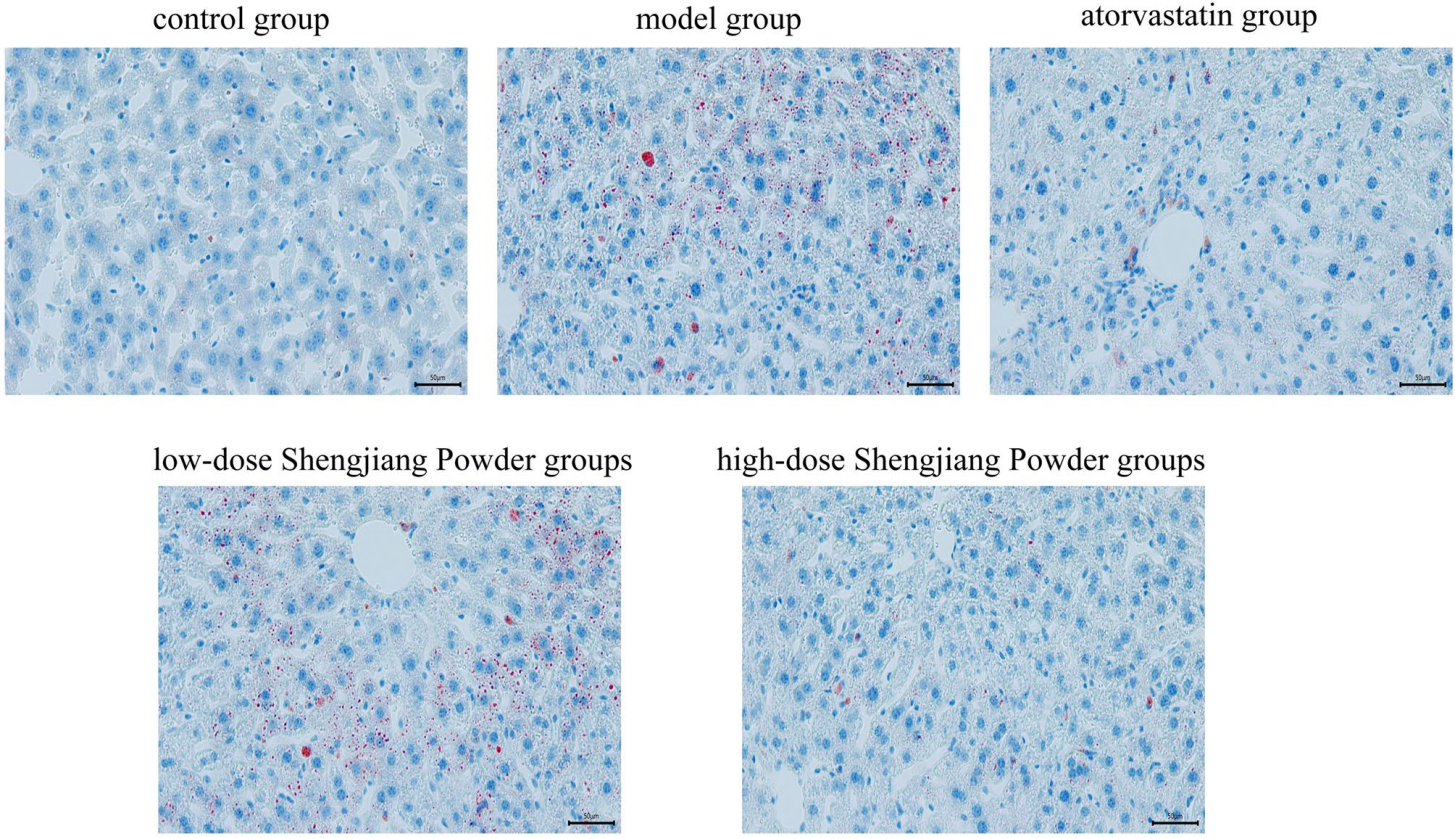
Microscopic changes in mouse liver with oil red O staining (40× magnification)

In order to assess the pathological effects of Shengjiang powder on fatty liver disease tissues, oil red O staining was used to observe the lesions of liver fat (Fig. 7), the results showed that the livers of control mouse were normal and almost oil droplet free while the livers of the model group showed a high number of oil droplets and a large size with the presence of lipid accumulation, and the number and size of the droplets in the atorvastatin group and the Shengjiang powder group were significantly reduced, and the Shengjiang powder showed dose effect, the higher the concentration, the better the effect.

### Shengjiang Powder inhibited the expression of inflammatory factors and activated the AMPK/mTOR signaling pathway

**Fig. 8:**
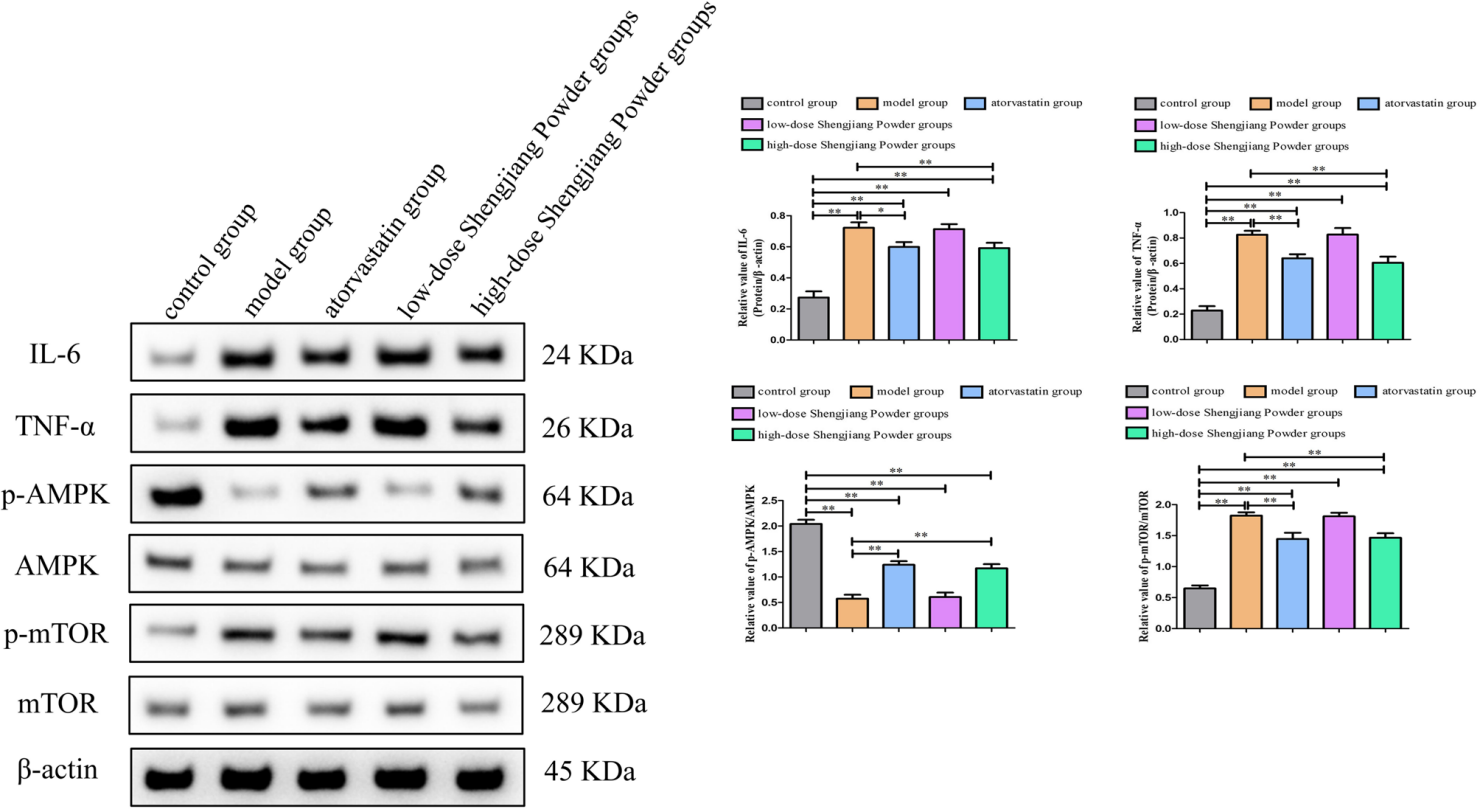
Shengjiang Powder inhibited the expression of inflammatory factors and activated the AMPK/mTOR signaling pathway. Western blot analysis revealed that high-concentration Shengjiang Powder downregulated the protein expression level of IL-6 and TNF-α, increased the *p*-AMPK/AMPK ratio, decreasing the *p*-mTOR/mTOR ratio. Statistical significance was determined as *p < 0.05, **p < 0.01

Compared to the model group, the protein expression levels of IL-6 and TNF-α were both suppressed in the high-dose Shengjiang Powder group(both *p* < 0.01) and atorvastatin group (IL-6 *p* < 0.05;TNF-α *p* < 0.01), but there was no difference compared in the low-dose Shengjiang Powder group(both *p* > 0.05). Compared to the model group, we found that high-concentration Shengjiang San and atorvastatin treatment both significantly increased the p-AMPK/AMPK ratio (both *p* < 0.01) in the liver, decreased the p-mTOR/mTOR ratio (both *p* < 0.01), but there was also no difference compared in the low-dose Shengjiang Powder group(both *p* > 0.05) (Fig. 8).

## Discussion

NAFLD is one of the most prevalent chronic liver diseases worldwide, with a global adult incidence rate reaching 25% [13], and an even higher rate of 29.2% among adults in China [14, 15]. Its progression encompasses simple non-alcoholic fatty liver and non-alcoholic steatohepatitis, which can advance to cirrhosis and ultimately hepatocellular carcinoma [16]. Numerous studies have demonstrated that NAFLD and various metabolic disorders, including atherosclerosis (AS), share a reciprocal causal relationship and mutually exacerbate each other’s development [17].

The traditional Chinese medicine formula “Shengjiang San,” highly esteemed by Chinese medicine practitioners, originates from the “Shanghan Wenyi Tiaobian.” Initially comprising six ingredients—Bombyx Batryticatus, Cicadae Periostracum, Curcumae Longae Rhizoma, Rhei Radix et Rhizoma, yellow rice wine, and honey—modern practice often utilizes the first four for medicinal purposes, hence the current composition of Shengjiang San refers to Bombyx Batryticatus, Cicadae Periostracum, Curcumae Longae Rhizoma, and Rhei Radix et Rhizoma. In this formula, Bombyx Batryticatus and Cicadae Periostracum detoxify and elevate the clear, while Curcumae Longae Rhizoma and Rhei Radix et Rhizoma dispel epidemic qi and descend the turbid. The combination employs both cold and warm properties, facilitating the interdependent ascending and descending movements, thereby achieving the effect of elevating the clear and descending the turbid. Modern pharmacological studies have demonstrated that Cicadae Periostracum, Curcumae Longae Rhizoma, and Rhei Radix et Rhizoma all possess lipid-lowering effects. Specifically, curcumin activates autophagy to regulate oxidative stress and inflammatory responses, improving fatty degeneration in mice with NAFLD and AS [18]. Modified Shengjiang San or its combination with other medications exhibits therapeutic effects on NAFLD complicated by AS. However, such research is mostly confined to simple observations of clinical efficacy, with a notable lack of studies investigating the application of the original Shengjiang San formula and its underlying mechanisms [19, 20]. This significantly constrains the exploratory development of Shengjiang San for the treatment of NAFLD complicated by AS. In summary, based on the theories of “simultaneous liver and heart disease” and “qi mechanism dysregulation,” and integrating the holistic perspective of Chinese medicine, we innovatively propose the “autophagy-qi mechanism dysregulation-simultaneous liver and heart disease” theory. From the angle of “treating the liver and heart together, and dispelling stasis to dredge collaterals,” we have selected the traditional Chinese medicine formula Shengjiang San for the treatment of NAFLD complicated by AS. Experimental validation is conducted to explore the therapeutic effects of Shengjiang San and its potential mechanisms, which is of great significance for seeking effective medications for the prevention and treatment of NAFLD complicated by AS.

In the treatment of atherosclerosis, statins are commonly used as anti-cholesterol ester medications, and clinical studies have reported their beneficial effects on non-alcoholic fatty liver disease (NAFLD) [21]. However, with the prolongation of treatment duration, the issue of drug-induced hepatorenal dysfunction caused by statins cannot be overlooked [22]. In this study, two mice in the atorvastatin group exhibited marked lethargy; one showed a significant decrease in lower limb mobility, and both serum and pathological data indicated evident liver function impairment. The other mouse had a notable increase in serum creatinine, suggesting renal dysfunction, potentially related to rhabdomyolysis. Such adverse effects were not observed in the Shengjiang San treatment group. Based on the experimental results, statins demonstrated overall better direct improvement in atherosclerosis (AS) compared to the Chinese herbal medicine group. However, the high-dose Shengjiang San group exhibited superior efficacy in treating AS-associated NAFLD compared to the atorvastatin group, without apparent abnormalities in liver and kidney function. Moreover, liver antioxidant stress capacity was significantly enhanced, leading to higher overall treatment benefits.

Inflammation and abnormal lipid metabolism are pivotal throughout the entire course of atherosclerosis (AS) and non-alcoholic fatty liver disease (NAFLD). Under the pathological stimulation of various inflammatory factors, pathways related to lymphangiogenesis are activated, leading to lymphatic hyperplasia [23–25]. However, excessive pathological lymphatic hyperplasia and remodeling may become the pathophysiological drivers of impaired lipid efflux and inflammatory accumulation [26]. The permeability of intercellular junctions in hepatic lymphatic endothelial cells is crucial for the outward transport of lipoproteins and lipid peroxides from the liver. Pathological remodeling, characterized by thickened lymphatic walls, impairs this clearance function, exacerbating intrahepatic lipid metabolism disorders. Traditional Chinese medicine (TCM), particularly those that promote blood circulation and dredge collaterals or strengthen the spleen and dispel turbidity, plays a positive role in regulating lymphangiogenesis by inhibiting its excessive proliferation and reducing inflammation progression [27–29]. We observed a significant increase in the number of hepatic lymphatic vessels, irregular growth morphology, and thickened edges in the model group. Compared to the model group, the TCM treatment group showed marked improvement, suggesting that Shengjiang San also exerts a certain inhibitory effect on the pathological generation of lymphatic vessels.

Upon activation, AMPK promotes mitochondrial biogenesis and autophagy, thereby clearing damaged mitochondria and oxidative products, and alleviating oxidative stress [30]. Additionally, AMPK enhances cellular antioxidant capacity by upregulating the expression of antioxidant enzymes such as SOD and CAT [31, 32]. Excessive activation of mTOR inhibits the autophagy process, leading to the accumulation of damaged mitochondria and oxidative products within cells, thereby exacerbating oxidative stress [33]. Furthermore, mTOR promotes the expression of inflammatory factors by regulating the activity of transcription factors such as nuclear factor-κB (NF-κB), further intensifying oxidative stress and inflammatory responses [34]. The AMPK/mTOR pathway plays a crucial role in the development and progression of atherosclerosis and fatty liver by modulating oxidative stress and inflammatory processes [35, 36]. We found that high-concentration Shengjiang San treatment significantly increased the p-AMPK/AMPK ratio in the liver, while decreasing the p-mTOR/mTOR ratio and the expression levels of inflammatory factors (IL-6, TNF-α), indicating that Shengjiang San inhibits oxidative stress and inflammation through the AMPK/mTOR pathway, thereby exerting a beneficial effect on fatty liver.

In conclusion, our experimental evidence preliminarily demonstrates that Shengjiang San is safe and effective in the treatment of AS combined with NAFLD, with comprehensive efficacy superior to statin therapy. Its underlying mechanism involves inhibiting oxidative stress and inflammation through the AMPK/mTOR pathway and regulating the number of locally generated lymphatic vessels, thereby exerting a therapeutic effect on fatty liver.

## Supplementary Information

Supplementary Material 1.

Supplementary Material 2.

Supplementary Material 3.

Supplementary Material 4.

Supplementary Material 5.

Supplementary Material 6.

Supplementary Material 7.

Supplementary Material 8.

## Data Availability

All of the data generated during this study were included in this article.
