# Supplementary figures and images for: Shengjiang Powder alleviates oxidative stress damage and fibrosis in mice with atherosclerosis concurrent with non-alcoholic fatty liver disease
Source: Hereditas. 2025 Nov 12;162:228. doi: 10.1186/s41065-025-00598-y (PMC12613652; doi:10.1186/s41065-025-00598-y)

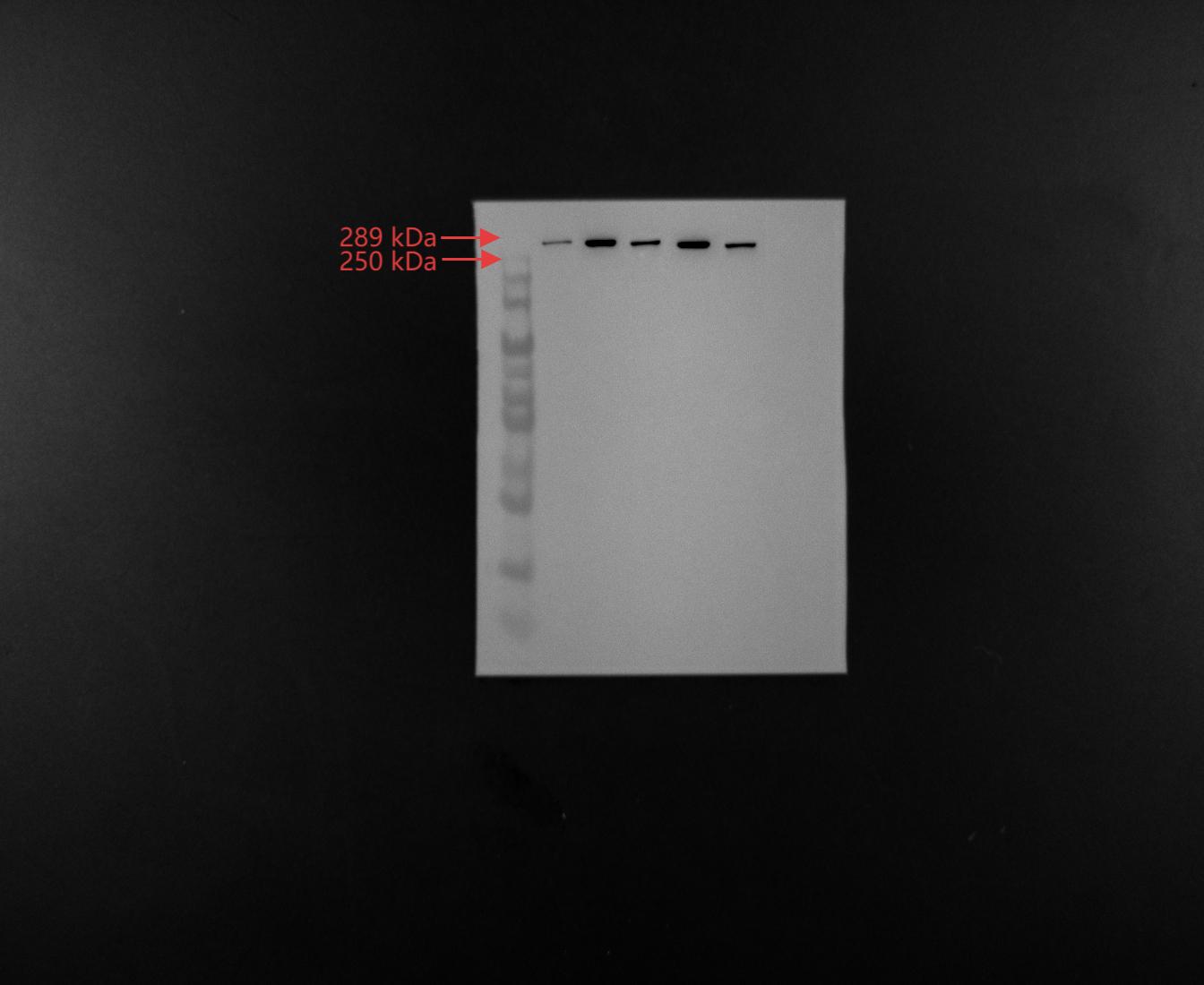

Supplement: Supplementary file 1 — Supplementary Material 1. [file 41065_2025_598_MOESM1_ESM.jpg]

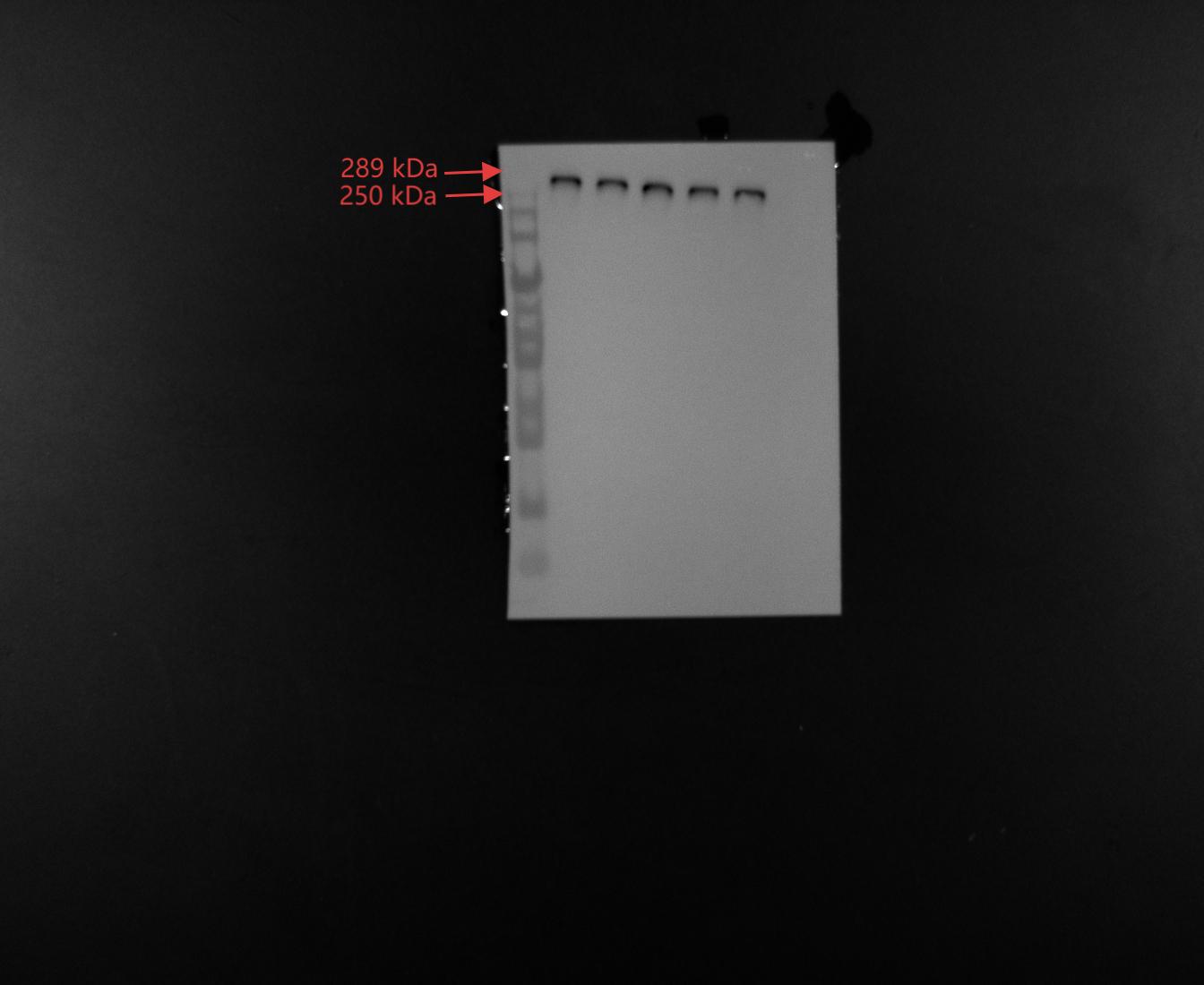

Supplement: Supplementary file 2 — Supplementary Material 2. [file 41065_2025_598_MOESM2_ESM.jpg]

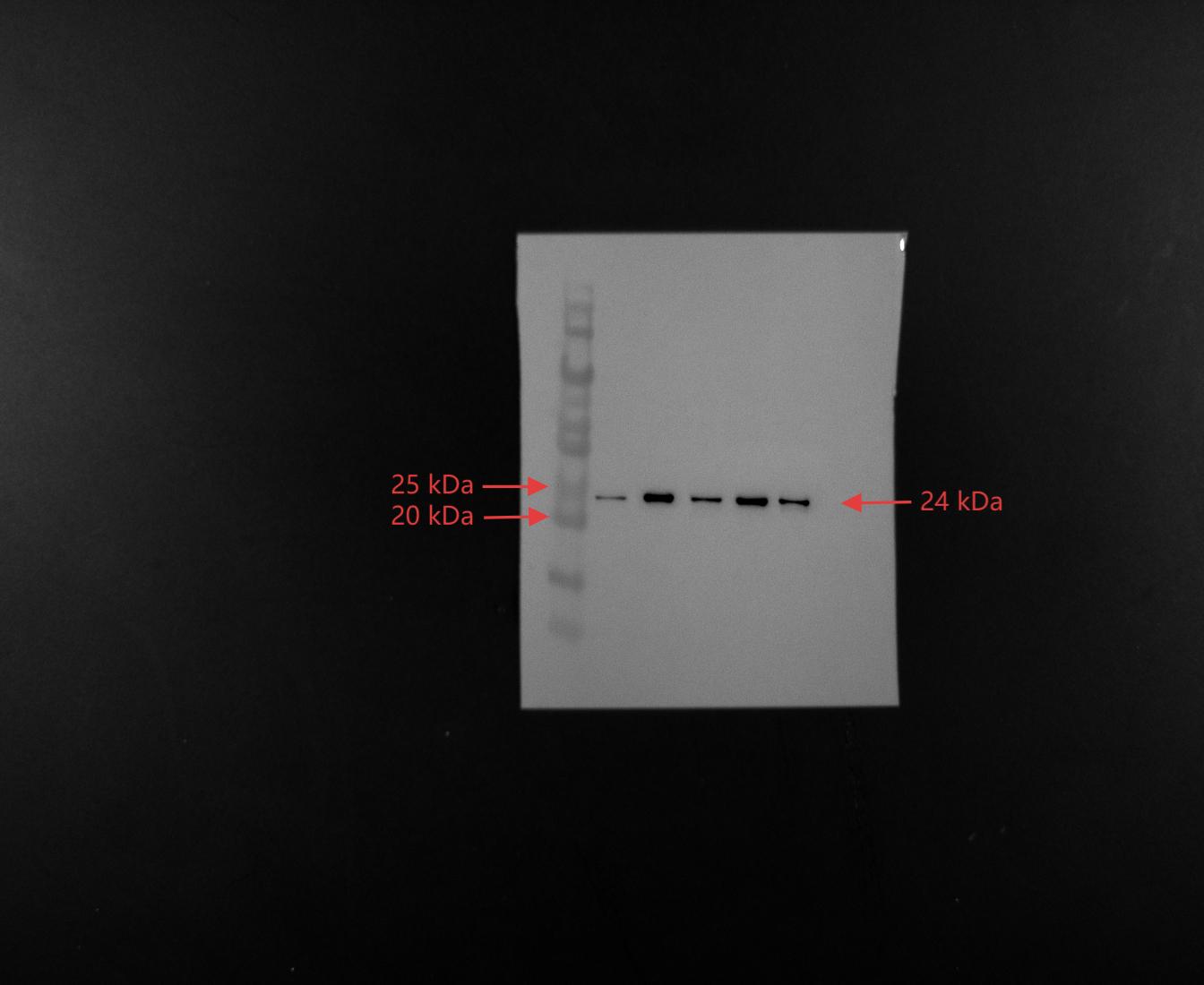

Supplement: Supplementary file 3 — Supplementary Material 3. [file 41065_2025_598_MOESM3_ESM.jpg]

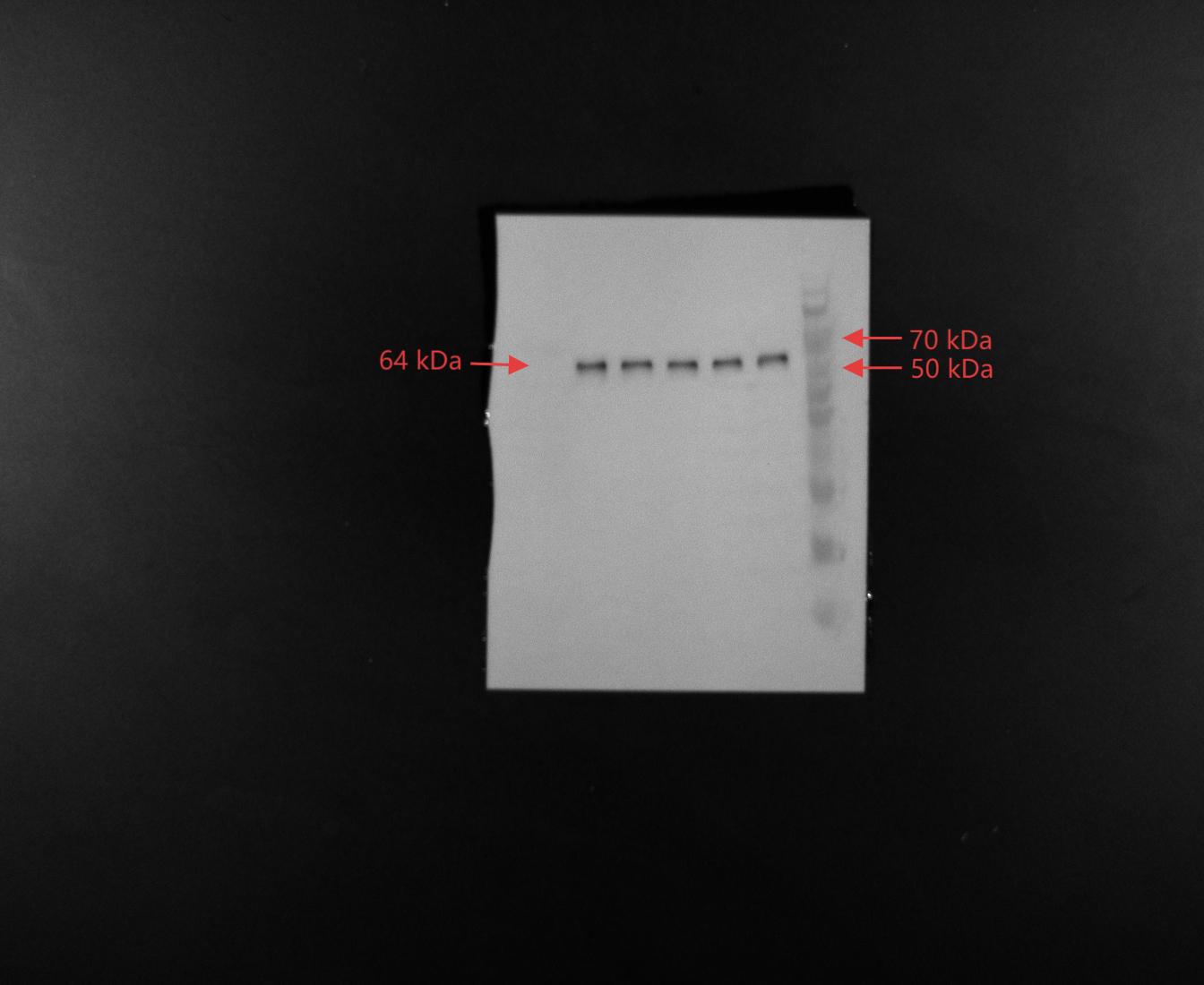

Supplement: Supplementary file 4 — Supplementary Material 4. [file 41065_2025_598_MOESM4_ESM.jpg]

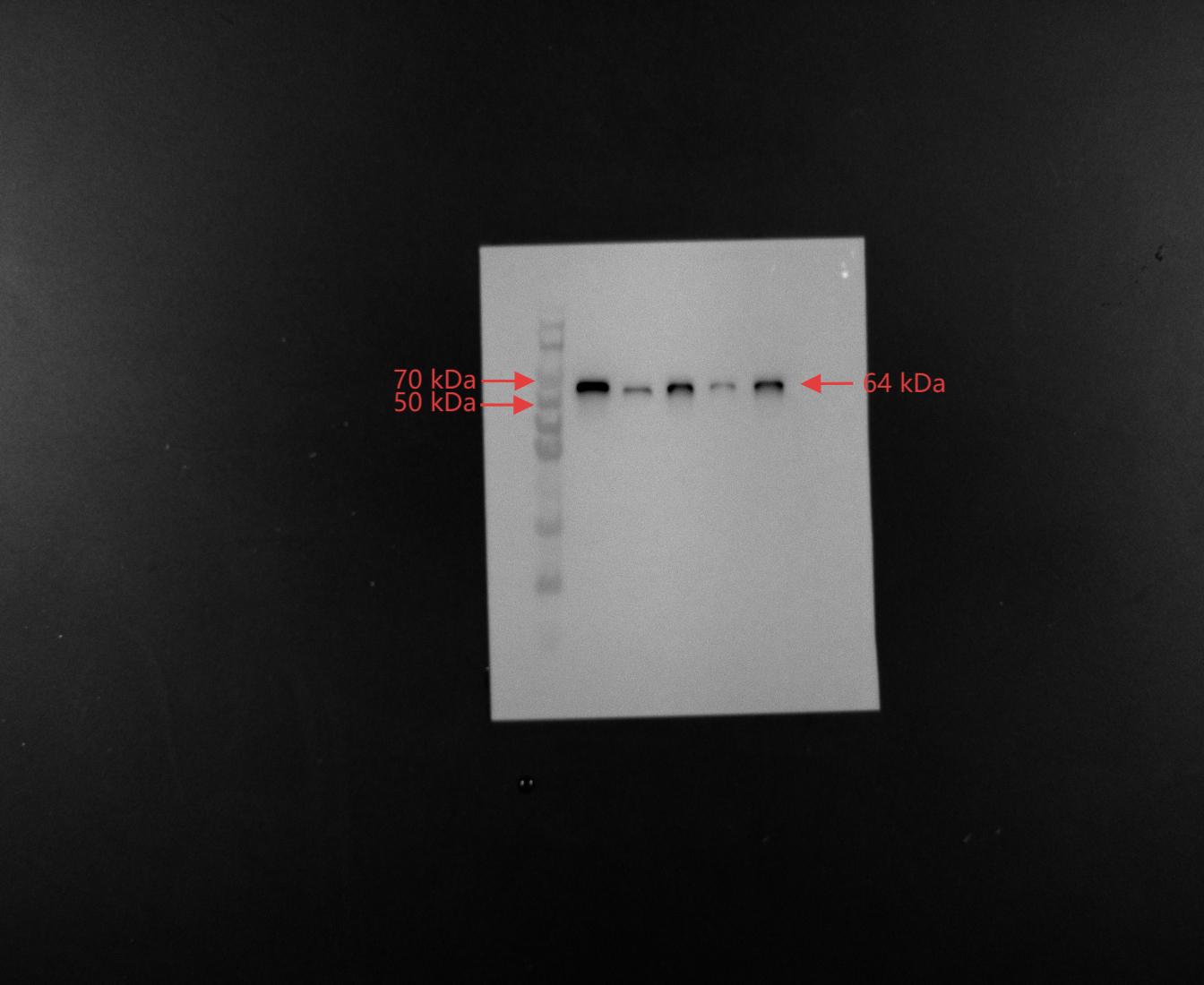

Supplement: Supplementary file 5 — Supplementary Material 5. [file 41065_2025_598_MOESM5_ESM.jpg]

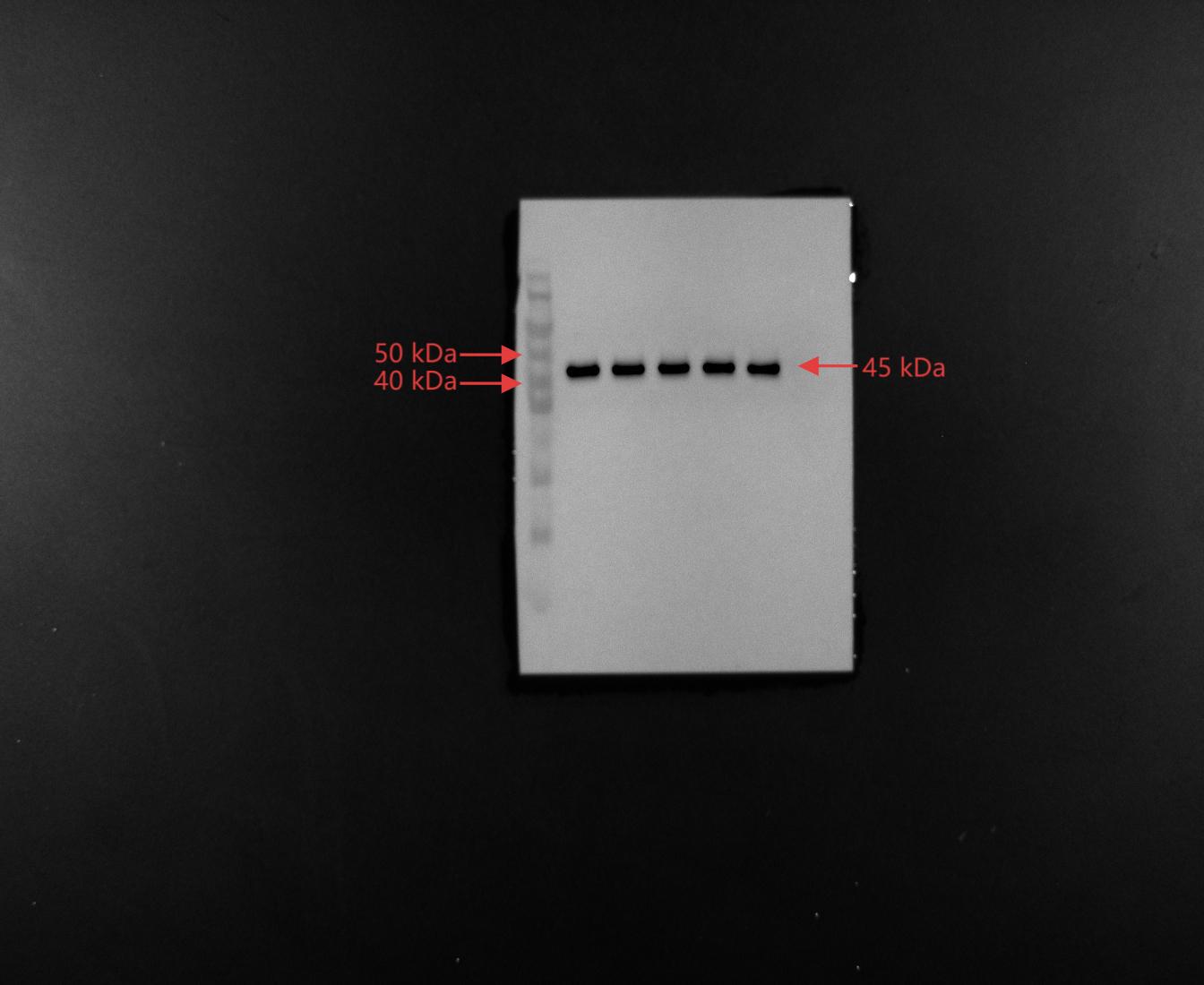

Supplement: Supplementary file 6 — Supplementary Material 6. [file 41065_2025_598_MOESM6_ESM.jpg]

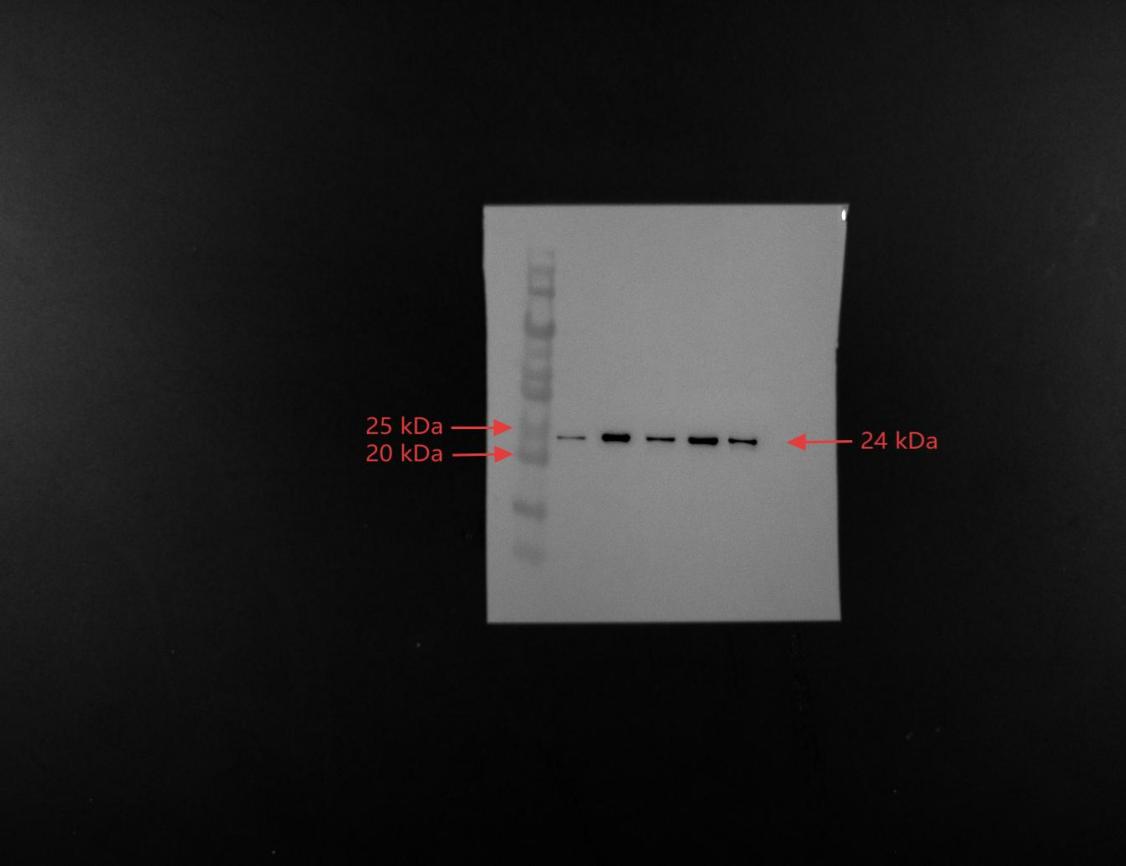


IL-6


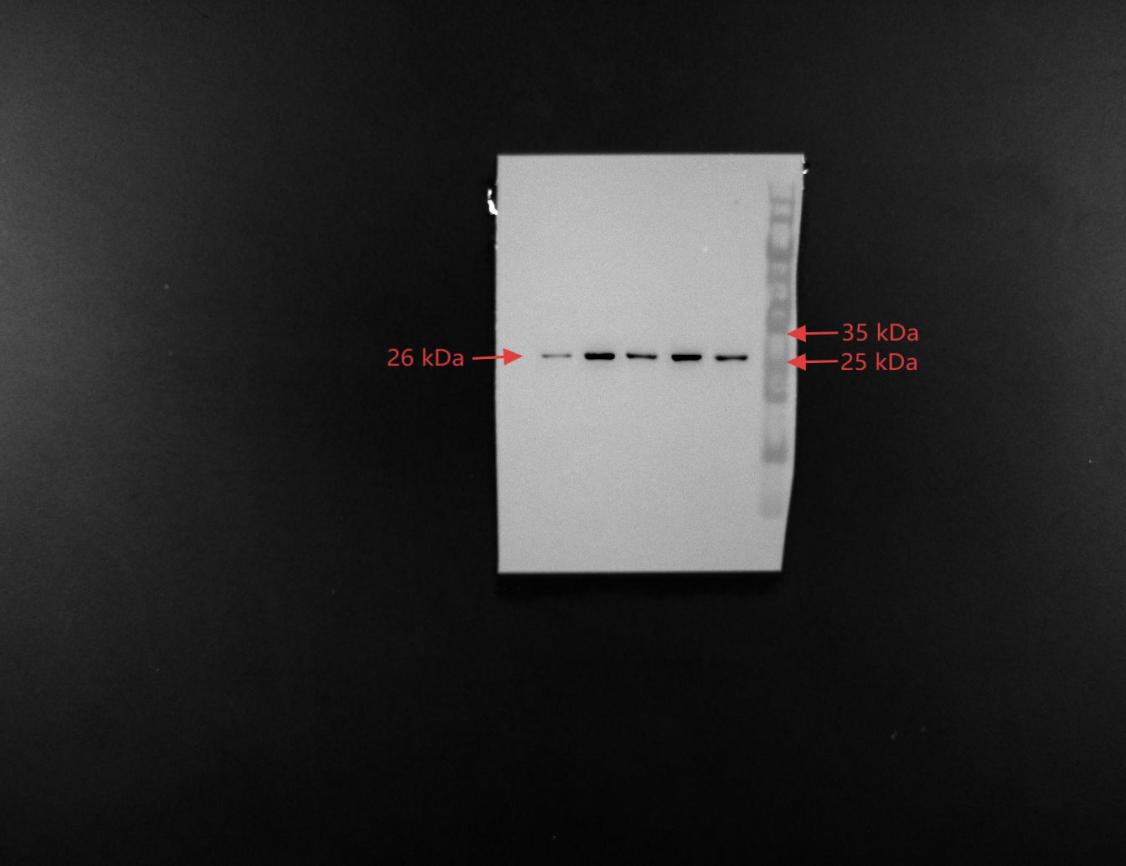


TNF-α


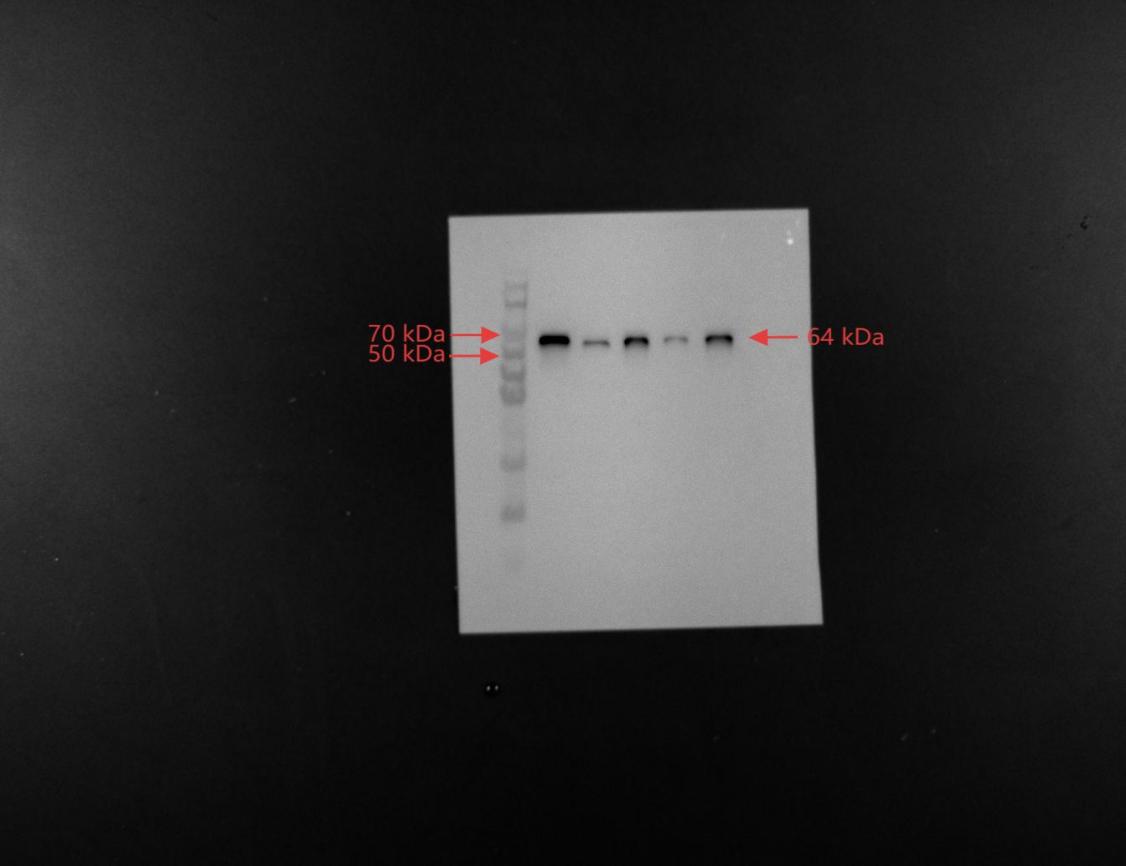


p-AMPK


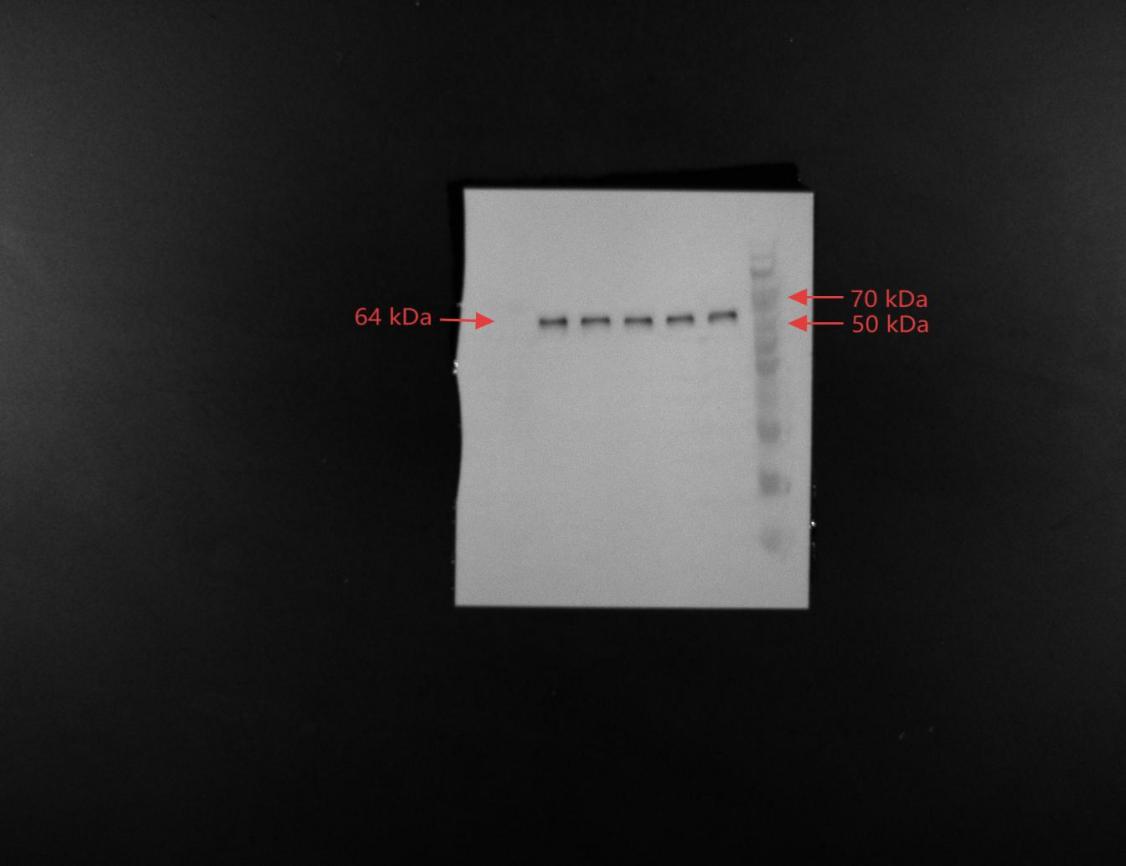


AMPK


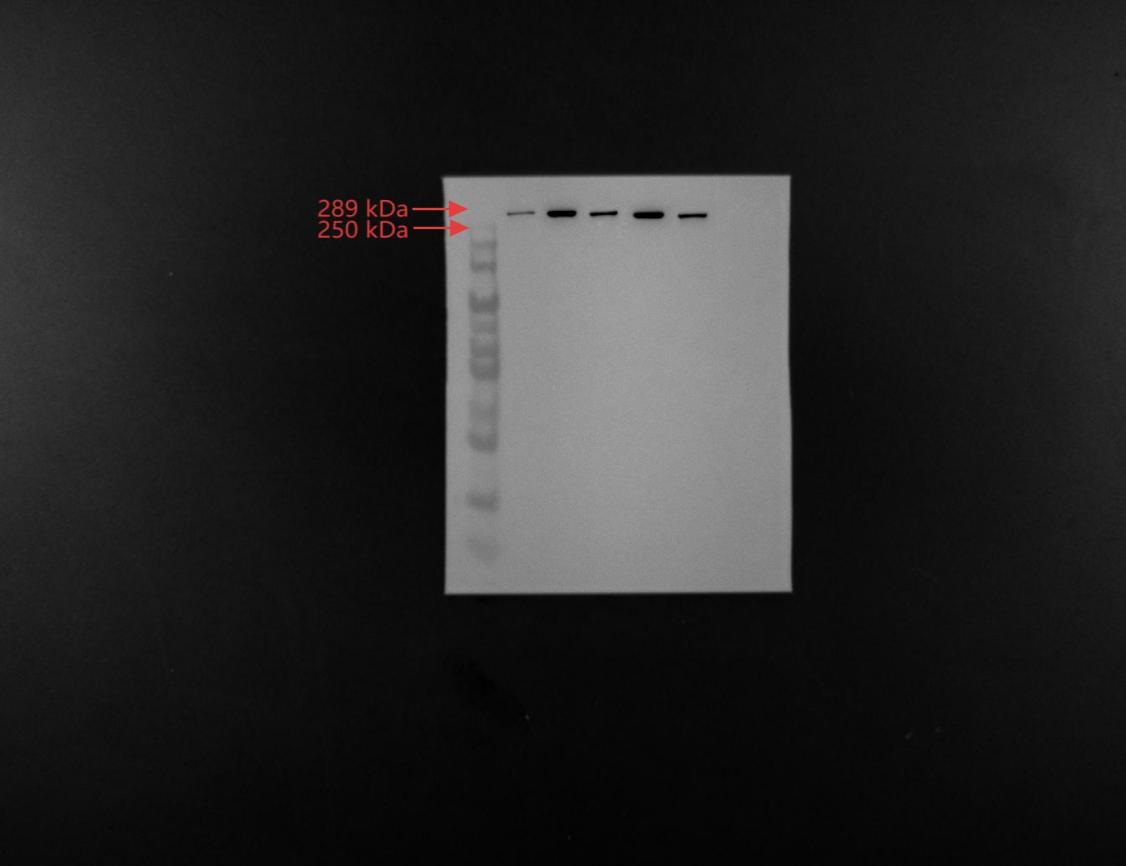


p-mTOR


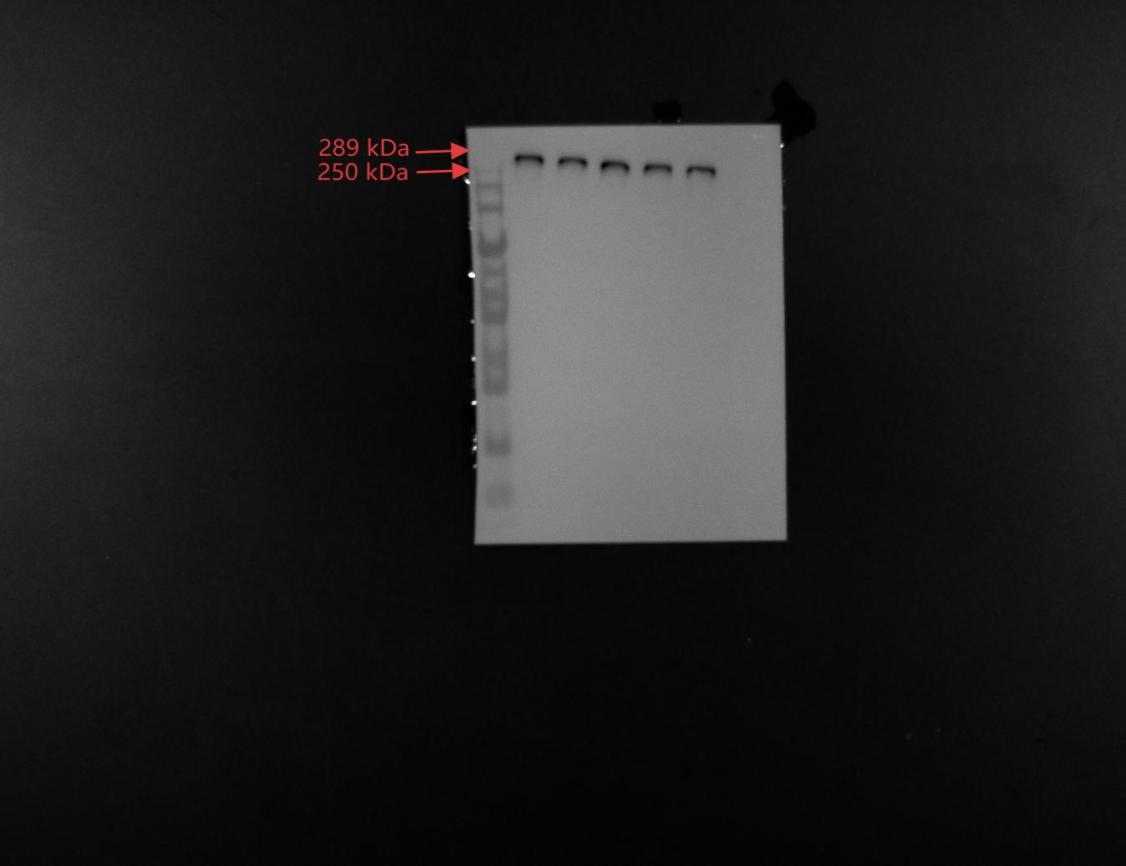


mTOR


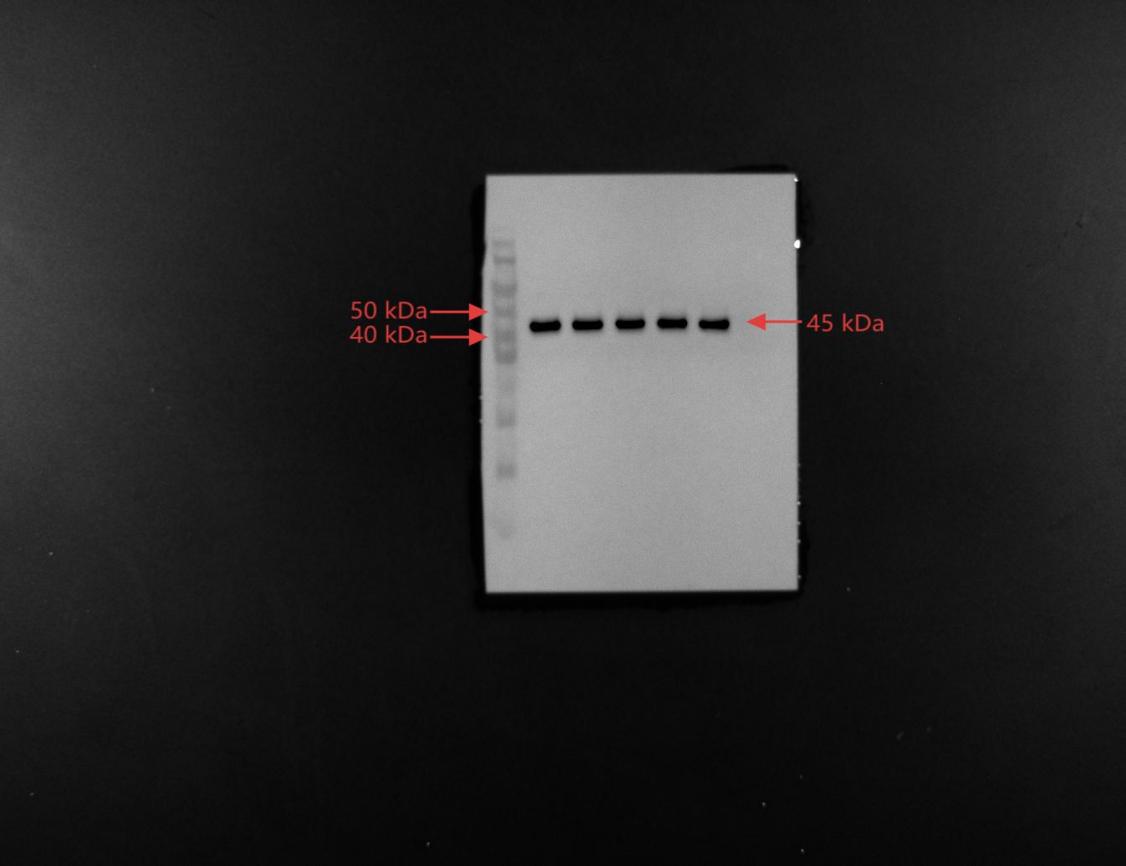


β-actin

Supplement: Supplementary file 7 — Supplementary Material 7. [file 41065_2025_598_MOESM7_ESM.docx]

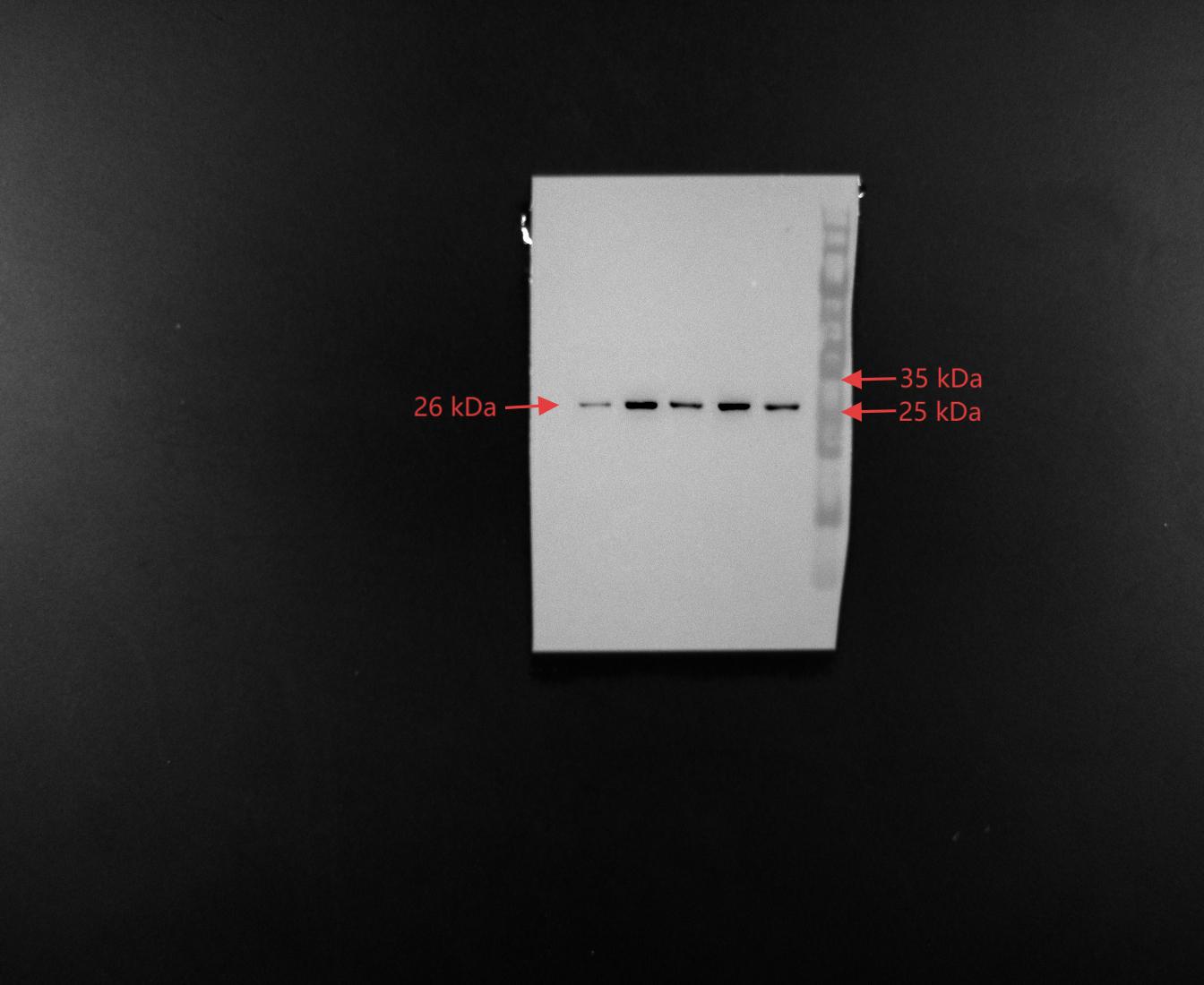

Supplement: Supplementary file 8 — Supplementary Material 8. [file 41065_2025_598_MOESM8_ESM.jpg]
